# Points to Consider on the Use of Medicines in Pregnancy Throughout the Product Lifecycle Based on Global Regulatory Guidance

**DOI:** 10.1007/s43441-024-00736-0

**Published:** 2025-02-22

**Authors:** Amalia Alexe, Keele Wurst, Leesha Balramsingh-Harry, Olatayo Apara, Nadezda Abramova, Osa Eisele, Maria Fernanda Scantamburlo Fernandes, Anju Garg, Birgit Kovacs, David Lewis

**Affiliations:** 1 University of Hertfordshire, Hertfordshire, UK; 2 GlaxoSmithKline, London, UK; 3 Hoffman LaRoche Ltd., Basel, Switzerland; 4 Merck & Co., Inc., Rahway, USA; 5 Merck Healthcare KGaA, Darmstadt, Germany; 6 Amgen Inc., Thousand Oaks, USA; 7 Eli Lilly, Indianapolis, USA; 8 Sanofi, Paris, France; 9 Boehringer Ingelheim Pharmaceuticals, Ingelheim, Germany; 10 Rue de la Tour de L’Ile 4, Genève, 1204 Switzerland

**Keywords:** Pharmacovigilance regulations, Patient safety, Pregnancy, Maternal health, Breastfeeding

## Abstract

The thalidomide tragedy of the 1960s led to restrictions and limitations in the participation of pregnant women in clinical trials. Despite the paucity of information on the safe and effective use of medicines in this population, most pregnant women are prescribed medications. A landscape assessment review of guidelines and legislation governing the use of medicines in pregnancy and during breastfeeding was conducted by the TransCelerate Pharmacovigilance Pregnancy and Breastfeeding Team. Insights from the landscape assessment review were compiled to identify important points to consider concerning the use of medicines in pregnancy throughout a product lifecycle. Four main areas were identified for consideration for use of medicines in pregnancy: (1) Product development considerations: Key points on the disease itself, the medicine characteristics, non-clinical and clinical development. (2) Interventional study considerations: Key aspects in enrollment of pregnant women in clinical trials and the follow-up requirements for such women. (3) Post-marketing considerations: Key elements in spontaneous case reporting of medicines exposure during pregnancy, implementation of appropriate risk management plans for medicines likely to be used in pregnancy. (4) Full lifecycle considerations: Activities required by regulators to ensure safety surveillance and maintenance throughout product lifecycle. There is a need for harmonized guidance on how to study the use of medicine in pregnancy. This paper addresses regulatory considerations, to aid in the planning and execution of research programs focused on developing medicines for use in pregnancy when permissible under established regulatory framework.

## Introduction

Globally, over 200 million women become pregnant each year [1]. In high income countries, 80% of women are prescribed medication during pregnancy and many more use additional over-the-counter medications [2]. Despite this fact, medication use during pregnancy is critically understudied. There is no legal or regulatory requirement for new drugs to be tested on pregnant women, leading to > 80% of pregnant patients routinely receiving therapies that have not been adequately studied in pregnancy [3]. Pervasive barriers stemming from past ethical, scientific, and legal concerns continue to impede research on drug safety and efficacy during pregnancy [4].

The thalidomide tragedy of the 1960s (that resulted in severe birth defects in thousands of children) led to restrictions which greatly limit pregnant women and women of child-bearing age’s participation in clinical trials [5]. The teratogenic effects of thalidomide also resulted in changes to United States (US) regulations. In 1977, the US Food and Drug Administration (FDA) excluded all “women of childbearing potential” from Phase I and early Phase II clinical trials due to fears that new medicines would harm fetuses [6]. However, in the past three decades, diversity in clinical trials became a policy priority in the US, advanced by federal agencies such as the Food and Drug Administration (FDA) Office of Women’s Health and the Society for Women’s Health Research (SWHR). Efforts have been made to promote inclusion of women of all ages, racial and ethnic groups, disabilities as well as those who are pregnant, lactating or breastfeeding in clinical trials [7]. FDA published a draft guidance for industry on scientific and ethical considerations for inclusion of pregnant women in clinical trials [8].

It is a global imperative to involve large numbers of women in research projects focusing on maternal health. In recent editorials, Riley [9] and Abbas-Hanif, et al., [10] underscored the need to include pregnant women in clinical trials and to invest in post marketing public health surveillance systems to monitor exposures to medicines during pregnancy and track maternal-fetal outcomes. Studies on pregnancy outcomes in HIV-infected women undergoing antiretroviral therapy [11] and the recent meta-analysis of COVID-19 vaccine use during pregnancy from Prasad, et al., [12] provide powerful examples of what can be achieved when there is an urgent need to protect maternal-fetal health. A meta-analysis of COVID-19 vaccination data found a greater likelihood of favourable outcomes from immunization during pregnancy for both the mother and baby and serves as a model of excellence for improving maternal healthcare.

Despite ongoing efforts by regulators mostly in the European Union (EU) and US to increase research involving pregnant women, knowledge on the safe use of medications during pregnancy remains limited [13]. It is important to note that the objective of many current guidelines is to protect pregnant patients and their fetuses by identifying and minimizing exposure to high-risk products. Inadvertently, this essential practice has also meant that guidelines advising how the industry can provide robust data to confirm the safety of low-risk products during pregnancy are not well developed [3, 14]. Consequently, there is a paucity of data to adequately inform healthcare providers, women, and their families about the benefits and risks of a medicinal product on pregnant women and fetuses via in-utero exposure. As such, many pregnant women with chronic health conditions are unwilling to continue maintenance therapy during pregnancy as they are uncertain of the drug’s safety for themselves and their unborn child [15].

Recognizing the importance of developing safe medications for pregnant and breastfeeding women, regulatory authorities across the EU, US, and United Kingdom (UK) have created guidance documents aimed at aiding research in this area. For example, in 2016, the Council for International Organisations of Medical Sciences (CIOMS) published the “International Ethical Guidelines for Health-related Research Involving Humans” recommending that, “pregnant women must not be considered vulnerable simply because they are pregnant” [16]. Similarly, the US Health and Human Services Office for Human Research Protection (45 CFR 46, 2019) removed pregnant women as an explicit example for a vulnerable population in 2019 [17].

The International Council for Harmonisation of Technical Requirements for Pharmaceuticals for Human Use (ICH) Efficacy Topic E21, which broadly addresses the inclusion of pregnant women in clinical trials, was released in 2023. However, in general, the scope of existing guidance documents is limited to specific timepoints in the drug development process and does not span the lifecycle of the drug.

In the EU, the European Medicines Agency has developed a pediatric investigation plan (PIP) aimed at ensuring the necessary data are obtained through pediatric studies to support the authorization of medicine for children [18]. Similarly, the ICH has the E11, R2 guideline on clinical investigations of pediatric medicinal products (E11, R1) which outlines the critical issues in the pediatric drug development process and the recommended approaches to study products in the pediatric population. Following the introduction of the PIP in 2007, there has been a more guided approach to the clinical development program for children. The proportion of clinical trials programs in children has increased by over 50% and has led to 260 new medicines or indications for children since the rules became effective [19]. A similar concept was recommended by the International Coalition for Medicines Regulatory Authorities (ICMRA) [20] and Medicines and Healthcare products Regulatory Agency (MHRA) [21] in the form of an obstetric/maternal investigational plan that could be implemented to guide the drug development in pregnant women. In the US, the Pediatric Research Equity Act gives the FDA the authority to require pediatric studies in certain drugs and biological products [22, 23]. The goal of these studies is to obtain pediatric labeling for the product [23]. Also in the US the Best Pharmaceuticals for Children Act encourages the pharmaceutical industry to perform pediatric studies to improve labeling for patented drug products used in children [24].The FDA created a guidance to provide recommendations to sponsors regarding the submission of an initial pediatric study plan (iPSP) and any amendments to the iPSP [25].Therefore, selected guidance regarding pediatrics is also summarized where points may be applicable to the evaluation of medicines in pregnancy.

TransCelerate BioPharma is a non-profit organization with more than 20 biopharmaceutical member companies that aim to streamline and accelerate the research and development of new therapies around the world. To meet the need for a comprehensive survey of global regulations and guidance on the use of medicines in pregnancy and during breastfeeding, the TransCelerate Pharmacovigilance Pregnancy and Breastfeeding Team [26] was formed in 2021 with the aim to propose solutions with a patient-centric approach that would facilitate the development of processes and tools for effective compliance with health authorities’ expectations in this area.

The purpose of this review is to provide a holistic summary of relevant global regulations and guidance that provide useful or necessary considerations for evaluating medicines in pregnancy and in women of childbearing potential (WOCBP) that may become pregnant, throughout the product lifecycle. This summary of key points is based on regulatory guidance and is intended for use by researchers when evaluating medicines in pregnancy throughout the product lifecycle to optimize their compliance with regulatory authority expectations. The topics considered in this review were limited to those covered in said regulations. As the points to consider for studying and including breastfeeding women in clinical trials may differ from those regarding women of childbearing potential or pregnant women, a separate points to consider (PTC) document for breastfeeding is warranted and, therefore, breastfeeding is beyond the scope of this review article.

*TransCelerate [or The Authors] provide these Points to Consider for informational purposes only. This Paper does not provide legal advice or guidance. Each marketing authorization holder (MAH) bears sole responsibility for its own compliance with all applicable laws or regulations*,* including identifying and construing all such laws and regulations. The views and opinions expressed herein are those of the authors; they do not necessarily reflect those of their affiliated companies.*

## Materials and Methods

**Fig. 1:**
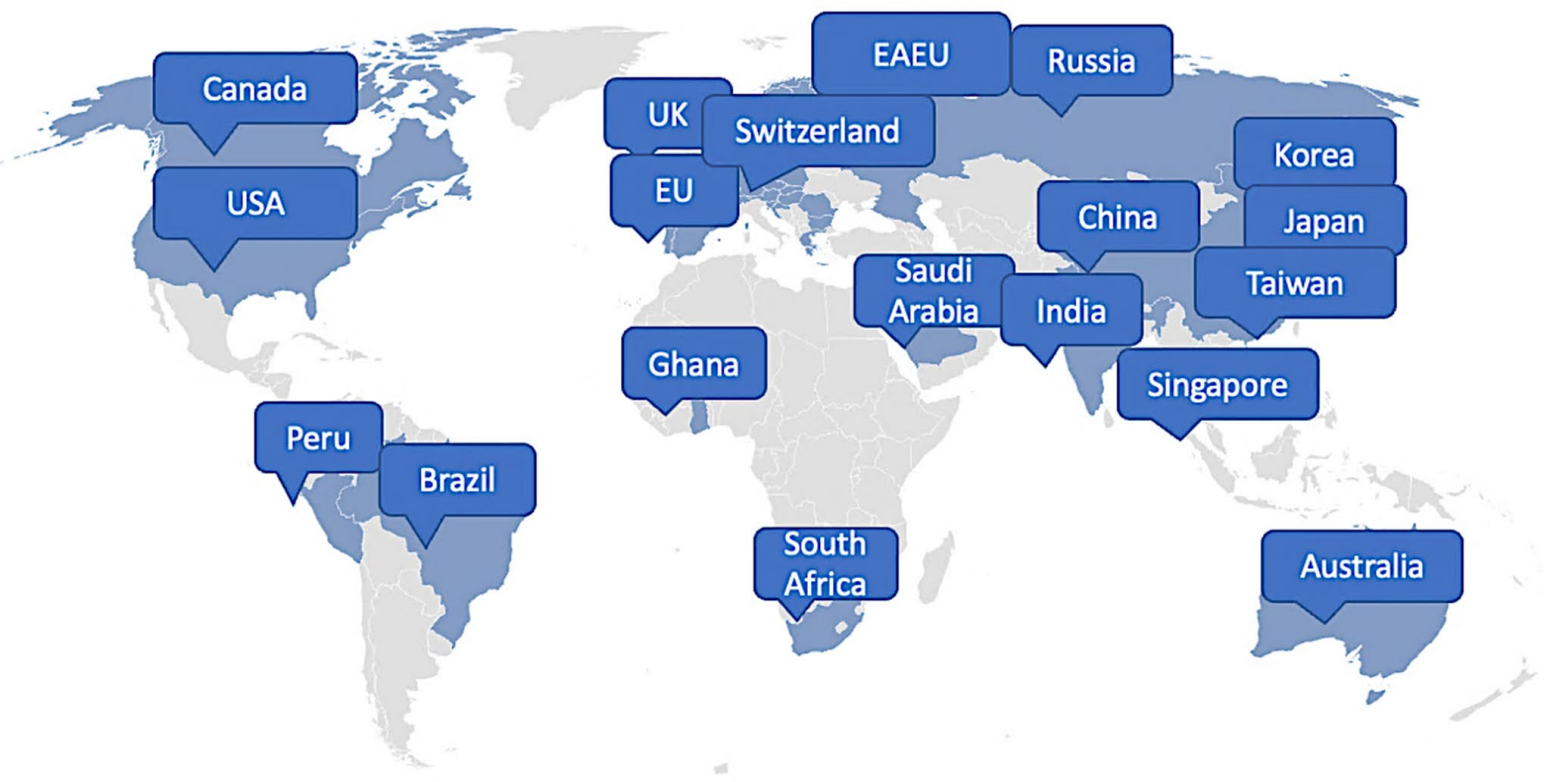
Countries and regions in scope for the assessment of regulatory landscape on the use of medicines in pregnancy and during breastfeeding. EAEU = Eurasian Economic Union, EU = European Union, UK = United Kingdom, USA = United States of America

The TransCelerate Pharmacovigilance Pregnancy and Breastfeeding Team conducted a comprehensive review [27] of approved guidelines and legislations governing the use of medicines in pregnancy and during breastfeeding that were available as of March 2022. The review encompassed international guidelines as well as national or regional guidelines in select countries (Fig. 1). Selection was based on public accessibility guidelines or regulations, their alignment with ICH standards, and availability of English translations for non-English texts. Regulations and guidelines that contain specific instructions for handling pregnancy and breastfeeding information were included in the review, while general safety regulations with no reference to pregnancy or breastfeeding were excluded.

ICH regulations and CIOMS guidance served as comparison points for all national or regional guidelines and regulations. Information gathered from various initiatives across private consortia, health authorities, and academia were included in the comprehensive review to provide a picture of the current guidelines, regulations, and initiatives on the use of medicines in pregnancy and while breastfeeding.

Insights from the review on pregnancy regulations were compiled and summarized in the complete “Points to Consider Concerning the Use of Medicines in Pregnancy throughout the Product Lifecycle” report document [28]. As the considerations for studying and inclusion of breastfeeding women may differ from those regarding WOCBP or pregnant women, these were not addressed in this current PTC document. Observations are presented by pharmacovigilance practice topic, spanning from clinical development through the product lifecycle. Where reference is made to WOCBP within the review, it should be noted that within this population, the focus is on pregnant women. Contraception legislation and guidance were not summarized in this review article; however, a proposed decision tree for contraception and pregnancy testing can be consulted within the TransCelerate Common Protocol Healthy Volunteer Library v08, Sect. 5.1 [29].

## Results

**Fig. 2:**
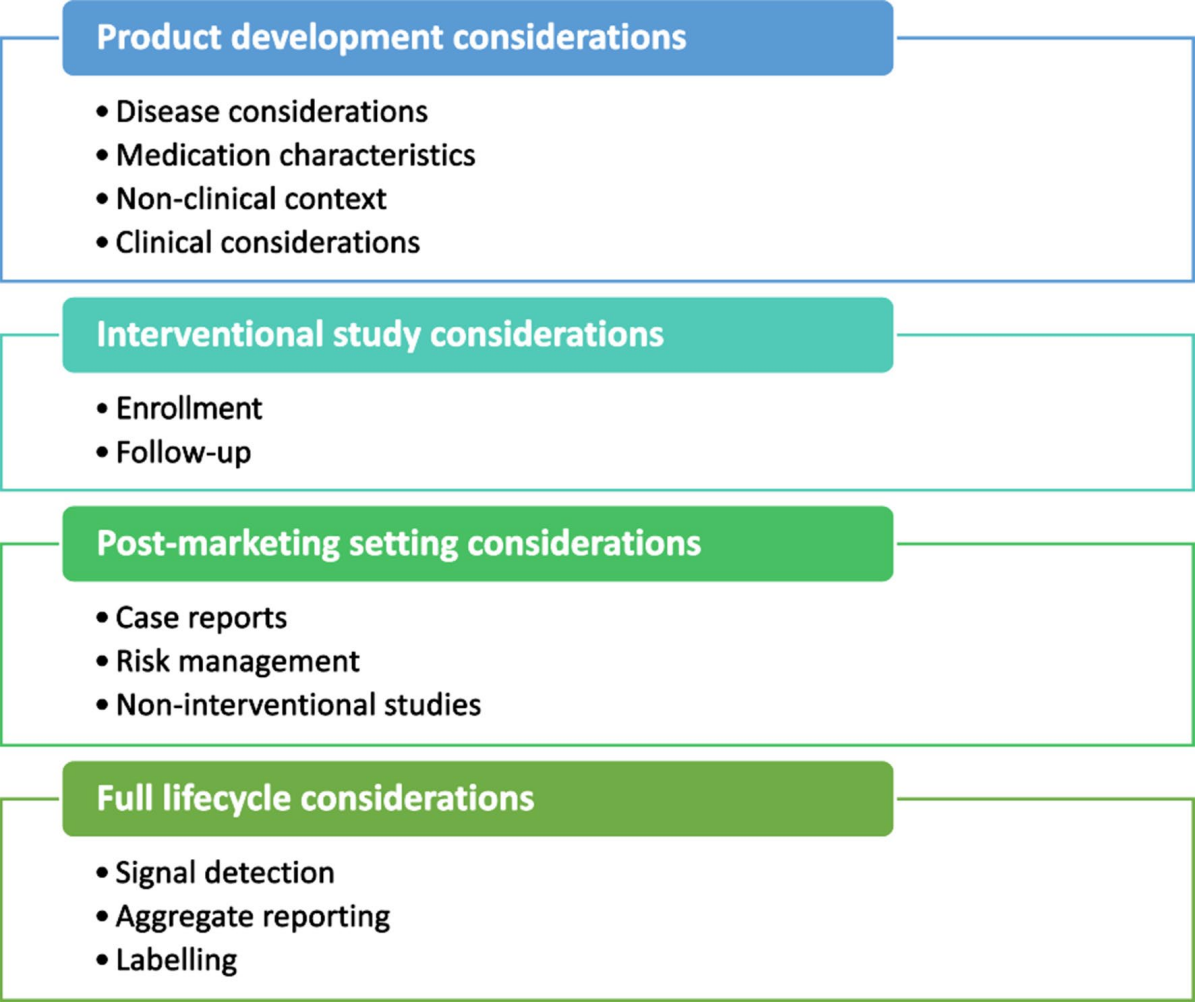
Areas of consideration from regulatory guidance documents on safety in pregnancy comprise of four main areas: product development, aspects relevant to interventional studies, requirements relevant to the post-marketing setting, and elements applicable to the full lifecycle

Regulatory guidance documents on safety in pregnancy include four main areas for consideration: Product development, aspects relevant to interventional studies, requirements relevant to the post-marketing setting, and elements applicable to the full lifecycle (Fig. 2). These areas were addressed in separate sections of the TransCelerate “Points to Consider Concerning the Use of Medicines in Pregnancy throughout the Product Lifecycle (Based on the Regulatory Guidance across the Globe)” report document [28]; the product development section of this report document addresses considerations for how and when to include pregnant women in the drug development process [28]. The interventional study considerations include details regarding international regulatory guidelines relating to enrollment in clinical trials and follow-up for clinical trials. Post-marketing study setting considerations include regulatory and international guidelines’ requirements for case reports, risk management, and non-interventional studies. The full lifecycle considerations section includes important aspects related to signal detection, aggregate reports, and labelling regulations.

### Product Development Considerations

#### Disease Considerations

Guidance on pediatric studies from both the US iPSP and the EU PIP includes disease presentation as an important consideration that may be applicable to the evaluation of medicines in pregnancy. Disease presentation includes a description of the etiology of the disease / condition, clinical manifestations, prognosis, prevalence, incidence, and epidemiology [18, 25]. Disease considerations include the context of current medical practice and available therapies [30]. Similar to pediatrics, if the disease is not prevalent in WOCBP, the development and post-marketing plans would differ from a disease that has a high prevalence in those populations.

#### Medication Characteristics

Medication characteristics such as the indication, mechanism of action, pharmacokinetics (PK), pharmacodynamics, sites of action, and potential or expected side effects are important considerations in both the EU and US.

The potential differences in the metabolic and PK aspects for pregnant women must be considered and compared to the general population. The US guidance on inclusion of pregnant women in clinical trials also highlights the importance of understanding the PK of a molecule. Because of the extensive physiological changes associated with pregnancy, PK parameters may change, sometimes enough to justify changes in dose or dosing regimen. Therefore, FDA guidance provides that PK data in pregnant women should be collected during clinical trials to guide appropriate dosing [8].

#### Non-clinical Considerations

As mentioned in the US and EU pediatric guidance, findings from animal and reproductive toxicology studies are important in developing a medication. It is necessary to determine whether prerequisites to human, maternal administration exist [18, 25]. Appropriate animal models and reproductive toxicology studies should be considered when developing a medication for pregnant women [18, 25]. It is important to identify what is known from animal reproductive toxicology studies and pregnancy models and whether a medication affects the growth and/or maturation of the fetus. The relevance to humans from these findings should be considered.

Potential teratogenic and genotoxic characteristics of the medication should be considered. Global guidelines advise performing female reproduction toxicity studies and standard genotoxicity tests before testing a medication in pregnant women in clinical trials. Non-clinical safety studies should be performed before human clinical trials and marketing authorization for pharmaceuticals (ICH guideline M3(R2)) [31].

#### Clinical Considerations

Similar to guidance in the pediatric development programs, there is a need for an improved benefit risk profile in a new molecular entity being developed in the population as compared to the current standard of care of the condition of interest [9, 18, 25]. It is critical to take into account the expected benefit, expected improvement in safety profile, including adverse event profile, drug interactions, potential medication errors, and the availability of clinically relevant and new therapeutic knowledge for the use of the medicinal product in the population [18, 25]. These should also be taken into consideration when determining medications for clinical development in pregnant women.

There may be challenges in performing clinical trials with pregnant women and WOCBP depending on the medication. Therefore, it is critical to reflect upon the feasibility of studying the population and condition of interest in both the clinical and post-marketing settings [18].

There are additional considerations for a new drug or new indication, where there is anticipated or actual use of the drug in pregnant women. Research involving pregnant women should demonstrate potential health benefits to pregnant women or the fetus, and new therapies would be expected to improve pregnancy and/or fetal outcomes as compared to the existing therapy. Any potential benefit to the mother and the fetus should be weighed against possible risks to both the fetus and the pregnant woman.

Feasibility of performing clinical trials investigating the condition of interest should be deliberated. The data needed to support the design or initiation of studies in paediatrics in addition to what may be needed for a feasibility assessment are critical factors [11]. These considerations would also apply to studies in women of childbearing potential and pregnant women.

### Interventional Study Considerations

#### Enrollment in Clinical Trials

Current regulations acknowledge that enrollment of pregnant women in clinical trials is complicated as exposure to an investigational product may present risks to the mother and fetus. Findings from non-clinical and animal studies should inform initial human doses and duration of exposure [9].

For WOCBP included in clinical trials, ICH M3 R2 includes additional guidance to minimize and characterize the risk of unintentional exposure of the pregnant woman, thus of the embryo and fetus. Canadian guidelines further clarify that enrollment decisions should be based on careful risk-benefit evaluation and consider factors such as “the nature and severity of the disease, the availability and results of previous nonclinical data on pregnant and non-pregnant animals, and results from clinical data” [30].

#### Follow-up for Clinical Trials

According to ICH E8 [32] and CIOMS VI [33], if a pregnancy occurs during a clinical trial, follow-up should be conducted for the health of the fetus and the pregnancy outcome (e.g., pregnancy termination, type of birth). Monitoring the pregnancy of a woman whose male partner is a trial participant may be needed in certain situations, such as when there is evidence of a class effect or when there is new evidence from animal models [33]. The duration of the follow-up is not clearly specified in the regulatory guidelines. The Canadian Guidance Document [30] and CIOMS VI [33] provides that, when possible, monitoring the development of the newborn and long-term follow-up of a child should be considered.

### Post-Marketing Setting Considerations

#### Case Reports

Regarding case reports, the US, EU, Canadian, Australian, Singapore, South African health authorities, and ICH all require MAHs to collect as much pregnancy exposure information as possible.

Requirements differ when considering which cases qualify for expedited reporting or which cases need only be discussed in periodic safety update reports. However, regulations indicate pregnancy exposure information should be collected and discussed in the periodic safety update reports [34–39]. In general, ICH, US, EU, Canadian, and the Australian guidelines provide that all reports where the embryo or fetus may have been exposed to medicinal products should be followed up [35, 40].

#### Risk Management

Per the EU guidance, “additional pharmacovigilance (PV) activities in the risk management plan (RMP) should be taken in a risk proportionate manner, considerations regarding risk proportionality will differ for the populations of pregnant women and breastfeeding women. The objective of the risk mitigation measure (RMM) is to reduce any risk to the child as much as possible given the need for treatment in the mother.” For products with anticipated use in WOCBP, a reflection of current understanding of safety in pregnancy should be included in the summary of the safety specifications in the RMP. The RMP should specifically discuss the likelihood of the use of the medicine in pregnancy, and WOCBP considering the proposed indications, alternative treatment options, need for effective contraception, and complexities of changing treatment if use during pregnancy is to be avoided [41, 42].

#### Non-Interventional Studies

Most information on the outcomes of drug exposure in pregnant individuals still relies on data generated post-approval. The ICH and CIOMS recognize that additional studies in the post-market setting may be warranted for pregnant women [43, 44]. In most regions reviewed, there were no clear regulations or guidance on when to consider these post-marketing studies nor best practices for conducting them. In regions where regulatory guidance for post-authorization pregnancy safety studies exists (primarily US and EU), recommendations tended to diverge from one another, with the level of detail included varying significantly between countries/regions.

The US guidance includes recommendations for when a product developer should establish a pregnancy exposure registry [37]. EU guidance provides that epidemiological studies should be carried out using existing data sources (i.e., secondary data) and be designed in such a way as to minimize bias [45]. In contrast, the US generally considers secondary data sources (e.g., electronic data sources, such as insurance claims and electronic health record databases) as additional studies that complement data obtained from pregnancy registries [39].

### Full Lifecycle Considerations

#### Signal Detection

ICH [43], CIOMS [46], Australia [40], EU [38, 41, 47], Eurasian Economic Union (EAEU) [43], Saudi Arabia [31], Switzerland [48], and US [37] guidance and regulations mention signal detection in pregnant women. As per CIOMS [46] and EU [48] guidance and regulations, the methods for signal detection depend upon the product, indication, type of adverse outcome to be monitored, and magnitude of risk. In addition, possible signals of teratogenic effects are a significant safety issue and, per regulations in Australia, EU [35], Saudi Arabia [31], and Switzerland [49], may require timely notification to health authorities.

#### Aggregate Reports

Per ICH, EU [38, 41, 47] and India [49], pregnant or lactating women exposed during clinical trials or post-approval should be included as a special exposure population in both the development safety update report (DSUR) and periodic safety update report in the periodic benefit risk evaluation report (PBRER), also referred to as the periodic safety update report (PSUR). Whereas ICH E2C (R2) does not require a summary of pregnancy outcomes in the PBRER, Australia and EU do require a summary of pregnancy outcomes to be included in the PSUR.

#### Labelling Regulations

International labelling provisions for pregnant or lactating women are similar, but country-specific requirements can vary. In 2008, Europe implemented guidance on the risk assessment of medicinal products on human reproduction and lactation, entitled ‘From data to labelling’ [50]. The FDA initiated the Pregnancy Lactation Labeling Rule (PLLR), effective June 30, 2015, which includes descriptive language on adverse outcomes following drug exposure and specific disease states during pregnancy [51].

The FDA requires manufacturers to update their Prescribing Information (PI) when new information becomes available that causes the current PI to be inaccurate, false, or misleading [52]. Updates may include indications, uses, populations, dosages, safety information, or other information on how to use the medicine safely and effectively [52].

## Discussion

Robust research investigating drug safety during pregnancy is fundamental to addressing the barriers that exist for maternal populations requiring pharmaceutical therapy. Numerous initiatives worldwide (e.g., International Coalition of Medicines Regulatory Authorities, the European Network of Centres for Pharmacoepidemiology and Pharmacovigilance, the Task Force on Research Specific to Pregnant Women and Lactating Women, European Network of Teratology Information Services, the Innovative Medicines Initiative ConcePTION project, Association of the British Pharmaceutical Industry, Mother to Baby) are aiming to highlight opportunities and improve current safety of medicines used in pregnancy.

There is an acute need for harmonization of international guidance and regulations for fetal and maternal health. Currently, no end-to-end product development guidance exists to address key aspects throughout the product development cycle. While no investigational plan has been proposed or is required by health authorities, discussions with regulators regarding the development of a “maternal” or an “obstetric” investigational plan by sponsors are currently ongoing in some territories [20, 21]. Moreover, in May 2022, an ICH Working Group (ICH E21) was established with the objective to provide best practices to ensure appropriate inclusion and/or retention of pregnant and breastfeeding individuals in clinical trials (CTs) [53].

## Conclusion

The PTC report document, prepared by the TransCelerate Pregnancy and Breastfeeding Team, summarizes key regulatory considerations, aiming to aid in both the planning and execution of research studies involving WOCBP and pregnant women [28]. By using these key points to develop research protocols, collect and review data, and communicate results through the label, sponsors and MAHs will better support clinicians, WOCBP, and pregnant women to weigh the benefits and risks concerning pharmaceutical therapy during pregnancy.

## Data Availability

The basis of the data that support the findings in this manuscript can be accessed via the TransCelerate website: https://www.transceleratebiopharmainc.com/wp-content/uploads/2023/03/IGRPV_Points-to-Consider_3.8.2023.pdf.
